# Upregulation of Immune Process-Associated Genes in RAW264.7 Macrophage Cells in Response to* Burkholderia pseudomallei* Infection

**DOI:** 10.1155/2018/1235097

**Published:** 2018-06-04

**Authors:** Dongmei Peng, Feng Pang, Ruiyong Cao, Shu Zhu, Xiaojian Yang, Xin Nie, Zhenxing Zhang, Baobao Li, Haifeng Huang, Yaying Li, Guohua Li, Li Du, Fengyang Wang

**Affiliations:** College of Animal Science and Technology, College of Tropical Agriculture and Forestry, Hainan University, Hainan Key Lab of Tropical Animal Reproduction & Breeding and Epidemic Disease Research, Haidian Island, Haikou 570228, China

## Abstract

Melioidosis is a severe and fatal tropical zoonosis, which is triggered by* Burkholderia pseudomallei*. To better understand the host's response to infection of* B. pseudomallei*, an RNA-Seq technology was used to confirm differentially expressed genes (DEGs) in RAW264.7 cells infected with* B. pseudomallei*. In total, 4668 DEGs were identified across three time points (4, 8, and 11 hours after infection). Short Time-Series Expression Miner (STEM) analysis revealed the temporal gene expression profiles and identified seven significant patterns in a total of 26 profiles. Kyoto Encyclopedia of Genes and Genomes (KEGG) was utilized to confirm significantly enriched immune process-associated pathways, and 10 DEGs, including* Ccl9, Ifnb1, Tnfα, Ptgs2, Tnfaip3, Zbp1, Ccl5, Ifi202b, Nfkbia,* and* Nfkbie*, were mapped to eight immune process-associated pathways. Subsequent quantitative real-time PCR assays confirmed that the 10 DEGs were all upregulated during infection. Overall, the results showed that* B. pseudomallei* infection can initiate a time-series upregulation of immune process-associated DEGs in RAW264.7 macrophage cells. The discovery of this article helps us better understand the biological function of the immune process-associated genes during* B. pseudomallei* infection and may aid in the development of prophylaxis and treatment protocols for melioidosis.

## 1. Introduction

Melioidosis is a severe and fatal zoonosis most commonly found in tropical regions between the 20th parallels [1]. Melioidosis was first defined as a “glandular-like” disease by Alfred Whitmore and C.S Krishnaswami in 1911, but it was not until the latter half of 20th century that the importance of melioidosis to public health in Northern Australia and Southeast Asia was recognized [1, 2]. Latest research shows that the world is bearing a growing burden of melioidosis, with estimated 169,000 cases, around 50% mortality rate, across 34 countries each year [3]. Melioidosis is acquired via percutaneous inoculation, inhalation of aerosols, or ingestion of contaminated soil or water [4, 5]. The clinical syndromes of melioidosis include chronic abscesses, acute pneumonia, or septicemia [5, 6].

*B. pseudomallei* is an aerobic, motile, non-spore-forming, intracellular, Gram-negative bacillus. It readily grows on common laboratory media at 37°C and resists unfavorable circumstances such as extreme temperature, poor nutrition, acidic and alkaline conditions, and dehydration [4, 7].* B. pseudomallei* can resist many generally used antimicrobial reagents, including aminoglycosides, penicillins, cephalosporins, and rifamycins [4, 7, 8].

During* B. pseudomallei* infection, the bacteria utilize various virulence factors for survival and replication within both host macrophages and epithelial cells. The virulence factors identified to date in* B. pseudomallei* include capsule, lipopolysaccharide, flagella, pili, and effectors transported by type III, IV, and VI secretion systems [1, 9]. These virulence factors play important roles when* B. pseudomallei* interacts with host innate immune cells.

When invading into macrophage, bacteria can stimulate cell fusion and further induce the generation of multinucleated giant cells (MNGC). However, if intracellular bacterial reproduction reaches a key stage, the bacteria induce death of the host cells and are released to induce secondary infections [1, 10, 11].

To better understand the molecular mechanisms of host-bacterial interactions during* B. pseudomallei* infection, DNA microarray and transcriptome analyses have previously been performed using* B. pseudomallei*-infected murine liver and spleen tissue. The results indicated that the Toll-like receptor (TLR) 2 pathway has responsibility for initiating host defense responses to* B. pseudomallei* invasion, and the expression levels of genes encoding several other TLRs (TLR3, 4, 5, 6, and 7) were also modulated. Nucleotide-binding oligomerization domain (*Nod*) 1,* Nod2*, nucleotide-binding and leucine-rich repeat receptor family pyrin domain-containing (*Nlrp*) 2,* Nlrp3,* and class II transactivator (*CIIta*) were elevated upon infection. Dysregulation of these genes can activate caspase (*Casp*) cascades, inducing apoptosis and amplifying inflammatory responses to intracellular pathogens [12]. To identify host responses that are directly or indirectly regulated by* Burkholderia* invasion protein C (BipC), transcriptomic analysis of the liver and spleen tissues of infected mice was performed. The results demonstrated that BipC mainly targets cellular processes related pathways, which can modulate cellular trafficking [13].

Although macrophages played a critical role in controlling bacterial replication in the early stage of infection, until now, our understanding about how macrophages respond to* B. pseudomallei* infection was still limited. In this study, we used an RNA-Seq based approach to carry out systemic analysis of changes in mRNA level of RAW264.7 cells infected with* B. pseudomallei* at 0, 4, 8, and 11 hours postinfection, hoping to find more useful details about the interaction between* B. pseudomallei* and its host.

## 2. Materials and Methods

### 2.1. Cell Culture and* B. pseudomallei* Infection

RAW264.7 cell line was purchased from the Cell Bank of the Chinese Academy of Science (Shanghai, China) and maintained as previously described [14]. The* B. pseudomallei* strain used in this study, BPHN1, was isolated from a goat in Hainan Province, China. To carry out infection assays, RAW264.7 cell monolayers (~1 × 10^6^ cells/well) in 6-well tissue culture plates were infected with the bacterial inoculum at a multiplicity of infection (MOI) of 10. After incubation for one hour at 37°C incubator, the cells were rinsed three times with phosphate-buffered saline, and 2 mL of fresh DMEM containing kanamycin (250 *μ*g/mL) was added to each well. At 4, 8, or 11 hours postinfection, cells were rinsed three times with PBS and total RNA was extracted using Trizol reagent (Invitrogen, Carlsbad, CA) according to the manuals. The collected RNA was then used for RNA-Seq and quantitative real-time PCR (qRT-PCR) analysis.

### 2.2. mRNA Library Construction and Sequencing

Construction of mRNA library and sequencing were performed as described previously [15]. Briefly, total mRNA was enriched using Oligo (dT) beads (Epicenter, Madison, WI, USA), fragmented, and reverse-transcribed. Second strand cDNA was obtained using DNA polymerase I, RNase H. Then the cDNA fragments were purified using a QIAQuick PCR Extraction Kit (Qiagen, Hilden, Germany) and end-repaired; a poly(A) tail added and then ligated to Illumina sequencing adapters. The ligation products were finally sequenced using Illumina HiSeq TM2500 by Gene Denovo Biotechnology Co (Guangzhou, China).

### 2.3. Mapping and Normalization of Gene Expression Level

Clean reads were obtained by removing low quality raw reads following the basic filtering rules: removing reads containing adapters; removing reads containing more than 10% of unknown nucleotides (N); removing low quality reads containing more than 50% of low quality (Q-value ≤ 20) bases. Bowtie 2 was used to map reads to the ribosomal RNA (rRNA) database, to remove the rRNA-mapped reads [16]. The remaining reads for each sample were then mapped to the reference genome GRCm38.p4 (Ensembl release 84) using TopHat 2 (version 2.0.3.12) [17]. After mapping, Cufflinks was used to assemble transcripts, and Cuffmerge (part of the Cufflinks package) was used to merge the assembled transcripts with the reference annotation and track Cufflinks transcripts across multiple experimental groups [18]. FPKM method was used to normalize gene expression level [19].

The edgeR package (http://www.r-project.org/) was used to identify differentially expressed genes (DEGs) across experimental groups [20]. And the selected standards for DEGs were fold change ≥ 2 and FDR (false discovery rate) < 0.05 in comparisons.

### 2.4. Short Time-Series Expression Miner (STEM) Analysis

STEM software could cluster, compare, and visualize gene expression trends during a short time series [21].The expression data of R0, R4, R8, and R11 sample were normalized to 0; then, log2 (R4/R0), log2 (R8/R0), and log2 (R11/R0) were clustered by STEM software as previously described [15, 21]. The clustered profiles with p value < 0.05 were considered as significant profiles.

### 2.5. KEGG Database Analysis

To identify significantly enriched signal pathways and better understand the biological functions of DEGs, the KEGG database was used for pathway annotation as Kanehisa described [22].

### 2.6. qRT-PCR Analysis of mRNA Expression

To validate the expression of DEGs, the total RNA isolated from different samples was used for qRT-PCR analysis with specific primers designed and* Gapdh* was used as internal control as previously described [23].

### 2.7. Statistical Analysis

Statistically significant differences of each comparison were identified by Student's t-test, and p value < 0.05 was regarded as statistically significant. KEGG pathways (hypergeometric p value ⩽ 0.05) were selected as the significant enrichment pathways among all DEGs.

## 3. Results

### 3.1. Infection of RAW264.7 Cells by* B. pseudomallei*

**Figure 1:**
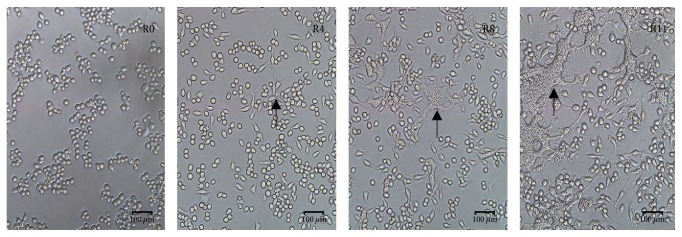
Cell fusion and formation of MNGC when the RAW264.7 cells were infected by* B. pseudomallei* (MOI = 10) at different time points. R0: RAW264.7 cells; R4: RAW264.7 cells at 4h.p.i; R8: RAW264.7 cells at 8h.p.i; R11: RAW264.7 cells at 11h.p.i. The black arrow showed the MNGC.

RAW264.7 cells were infected by* B. pseudomallei* at MOI = 10. At 4h postinfection (h.p.i.); cell fusion and multinucleate giant cells (MNGC) occurred; At 8h.p.i and 11h.p.i, there were more cell fusion and MNGC formation observed (Figure 1).* B. pseudomallei* could survive and proliferate within the macrophage cells and protect itself from host defense because the formation of MNGC inhibited the phagocytic ability and cell division activities of the host. The total RNA of R0, R4, R8, and R11 samples were collected for RNA sequencing and further analysis with uninfected RAW264.7 cells (R0 sample) as the negative control.

### 3.2. DEGs Statistics of* B. pseudomallei*-Infected RAW264.7 Cells

The raw RNA-Seq data of four samples was deposited into Sequence Read Archive (SRA) of National Center for Biotechnology Information (NCBI) with the accession number SRP115993. An average of 32 million clean reads per sample were obtained after removing low quality reads. The Q20 score was all above 96% and the mapping rate to reference genome of each sample varies from 87.12% to 88.98% (Table 1). All the data indicated that the quality of the RNA-Seq was excellent and could be used for further analysis.

**Figure 2:**
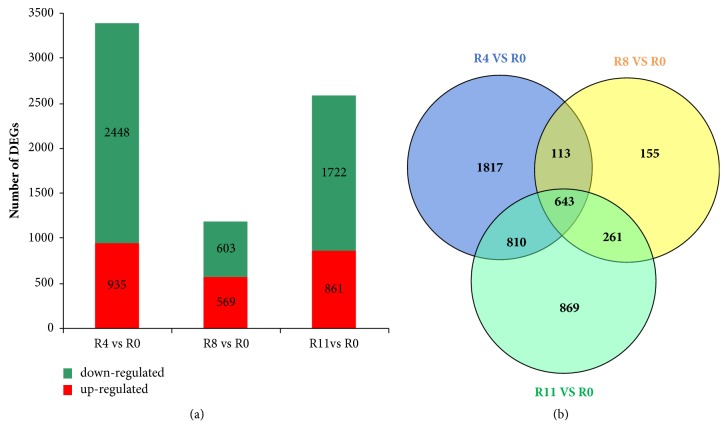
Large number of DEGs were induced by* B. pseudomallei* infection. (a) The graph showed the upregulated and downregulated DEGs from R4 versus R0, R8 versus R0, and R11 versus R0 comparisons, respectively. (b) The Venn diagram presented the number of DEGs from R4 versus R0, R8 versus R0, and R11 versus R0 comparisons.

A total of 2448, 603, and 1722 DEGs were downregulated among comparisons of R4 versus R0, R8 versus R0, and R11 versus R0, while 935, 569, and 861 DEGs were upregulated, respectively. Obviously, the number of downregulated genes was far more than that of the upregulated genes in R4 versus R0 and R11 versus R0 groups (Figure 2(a)). The results showed that* B. pseudomallei* infection changed the expression level of a number of host genes.

### 3.3. Identification of Upregulated Immune Process-Associated DEGs

**Figure 3:**
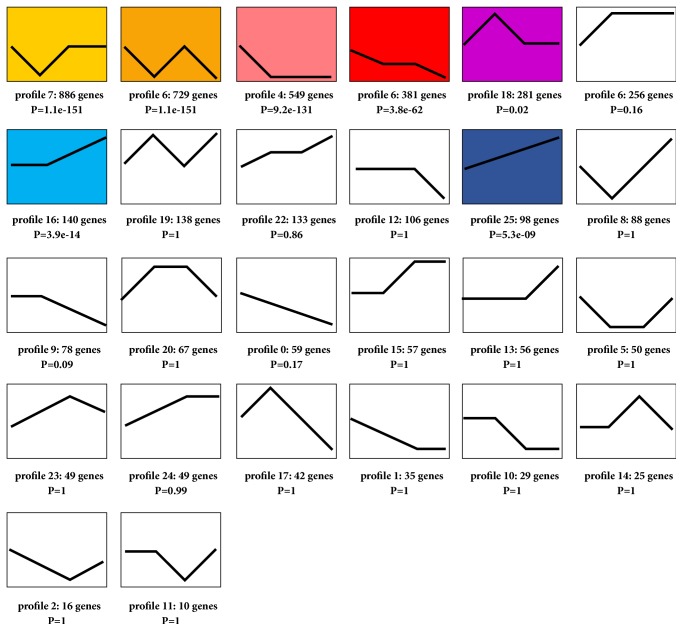
Trend analysis of the 4668 DEGs by STEM. The graph showed the total of 26 gene expression patterns in which seven colored boxes represented significantly enriched profiles (p value < 0.05). The number of DEGs and p value assigned to each profile were shown.

**Figure 4:**
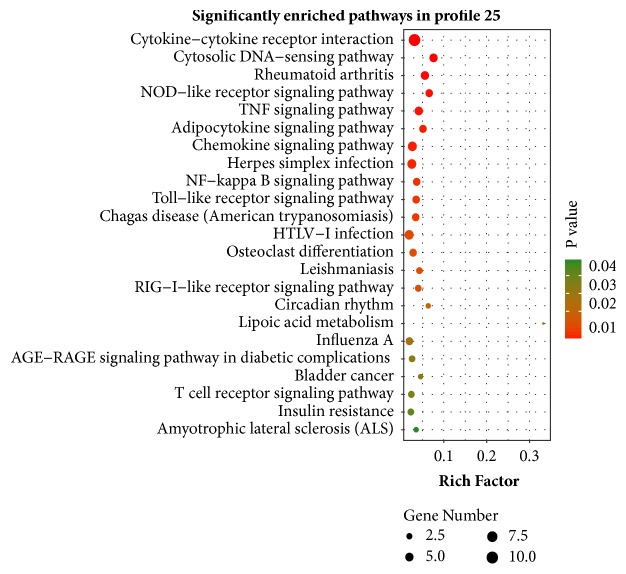
KEGG pathway enrichment analysis of DEGs in profile 25. Y-axis represented pathways, and X-axis represented rich factor (rich factor = the number of DEGs enriched in a pathway/the number of all genes annotated to the pathway).Color and size of each bubble represented enrichment significance and amount of DEGs enriched in a pathway, respectively.

The Venn diagram clearly identified a total of 4668 DEGs from R4 versus R0, R8 versus R0, and R11 versus R0 comparisons (Figure 2(b)). STEM clustering was performed to determine the exact gene expression patterns of the DEGs across the three paired groups. As a result, 26 clustered profiles were determined and seven profiles (profiles 7, 6, 4, 3, 18, 16, and 25) were significantly enriched with hypergeometric p value < 0.05 (Figure 3). DEGs from profile 25 displayed a time gradient rising trend. KEGG cluster analysis for the 98 DEGs in profile 25 suggested that 49 DEGs with pathway annotation were mapped to 23 significant pathways (*P* < 0.05) (Figure 4), which included cytokine-cytokine receptor interaction, cytosolic DNA-sensing pathway, TNF signaling pathway, chemokine signaling pathway, NF-kappa B signaling pathway, Toll-like receptor signaling pathway, and NOD-like receptor signaling pathway. Among them, 16 DEGs were mapped to eight immune process-associated pathways (Table 2). After removing overlapping and low-expression DEGs (FPKM< 10 at all four time points), 10 DEGs were obtained.

**Figure 5:**
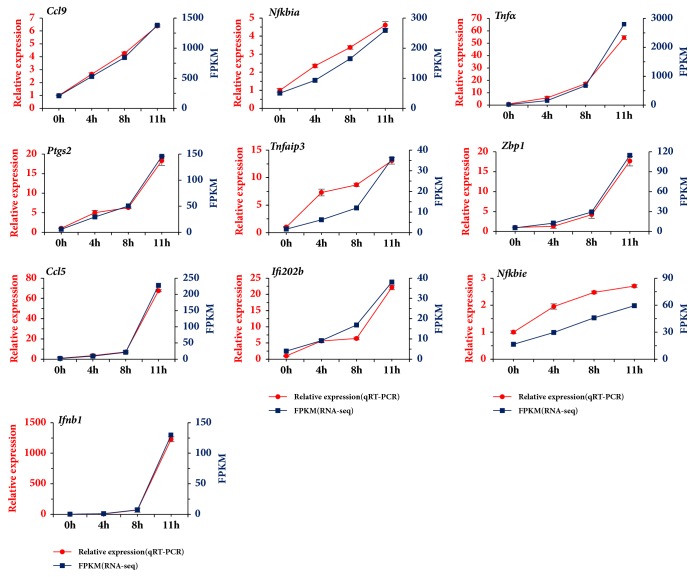
Validation of 10 DEGs by qRT-PCR. X-axis represented different time points postinfection. The left Y-axis represented the relative mRNA expression level of the DEGs by qRT-PCR, while the right Y-axis represented the FPKM of the DEGs from RNA-Seq. Data from qRT-PCR were means of three independent replicates and bars represented SD.

Specific primers for the 10 DEGs were designed (Supplementary Table 1), and qRT-PCR were performed with* Gapdh* as an internal control. The results indicated that the 10 DEGs, chemokine (C-C motif), ligand 9 (*Ccl9*), interferon beta 1, fibroblast (*Ifnb1*), tumor necrosis factor-alpha (*Tnfα*), prostaglandin-endoperoxide synthase 2 (*Ptgs2*), tumor necrosis factor, alpha-induced protein 3 (*Tnfaip3*), Z-DNA binding protein 1 (*Zbp1*), chemokine (C-C motif) ligand 5 (*Ccl5*), interferon activated gene 202b (*Ifi202b*), nuclear factor of kappa light polypeptide gene enhancer in B cells inhibitor, alpha (*Nfkbia*), and nuclear factor of kappa light polypeptide gene enhancer in B cells inhibitor, epsilon (*Nfkbie*), were all upregulated (Figure 5 and Supplementary Tables 2–4). The results of qRT-PCR analysis corresponded to that of RNA-Seq screening.

## 4. Discussion

Common hosts of* B. pseudomallei* include human, nonhuman primate, equid, goat, and rodent. Accumulating evidence indicated that melioidosis is transmitted by rodents including mouse [24]. In the study, RAW 264.7, a transformed murine macrophage cell line, was used as the infection model* in vitro*. During the early phase of* B. pseudomallei* infection, macrophages kill bacterial cells through the production of nitric oxide. This activity depends on INF-*γ*, which is originated from TCR-gamma delta T cells, CD8^+^ cells, and natural killer cells [25].

STEM software can cluster, compare, and visualize gene expression datasets during a short time series and compare the expression trends of these genes under different experimental conditions [21]. KEGG integrates genomic, chemical, network information databases and associated software [22, 26, 27]. In the study, we identified 26 gene expression profiles using the STEM software. After KEGG pathway analysis, seven significant enrichment profiles were acquired. In profile 7, DEGs with pathway annotation were mainly mapped to pathways in cancer, HTLV-I infection, thyroid hormone signaling pathway, focal adhesion, and AMPK signaling pathway. In profile 6, DEGs with pathway annotation were mainly mapped to cell cycle, inositol phosphate metabolism, regulation of actin cytoskeleton, and phosphatidylinositol signaling system. In profile 4, DEGs with pathway annotation were mainly mapped to lysosome, endocytosis, calcium signaling pathway and phosphatidylinositol signaling system. In profile 3, DEGs with pathway annotation were mainly mapped to pathways in cancer, cell cycle, fanconi anemia pathway, and hepatitis B. In profile 18, DEGs with pathway annotation were mainly mapped to ribosome, oxidative phosphorylation, Parkinson's disease, Huntington's disease, Alzheimer's disease, and nonalcoholic fatty liver disease (NAFLD). In profile 16, DEGs with pathway annotation were mainly mapped to influenza A, hepatitis C, herpes simplex infection, and measles (Supplementary Figure 1). Differently, in profile 25, 49 DEGs with pathway annotation were mainly mapped to cytokine-cytokine receptor interaction, cytosolic DNA-sensing pathway, TNF signaling pathway, chemokine signaling pathway, NF-kappa B signaling pathway, Toll-like receptor signaling pathway, and NOD-like receptor signaling pathway. Compared to the other six gene profiles, profile 25 contained many immune-associated pathways, and that is why we select it for further research, as it can help us to improve the understanding about the interaction between host cells and* B. pseudomallei.* 10 genes from profile 25 were confirmed using qRT-PCR assays, which suggested that the research strategy was feasible and effective.

Previous research of* B. pseudomallei* infection reported the upregulation of* Tnfα* and* Ptgs2*, two of the 10 DEGs identified in the current study [6, 28]. During* B. pseudomallei* infection, NF-*κ*B is activated, leading to the translation of IFN-*γ*, IL-6, IL-10, IL-1*β*, keratinocyte chemoattractant (CXCL1), TNF-*α*, and other proinflammatory cytokines. Activation of macrophage by* B. pseudomallei* also leads to generation of TNF-*α* [6]. In* B. pseudomallei*-infected THP-1 human monocytic leukemia cells, the upregulation of various inflammatory genes, including* Tnfα* and* Ptgs2* (the corresponding protein of which is also known as cyclooxygenase, COX-2), was observed [28]. These proinflammatory cytokines, including TNF-*α,* can enhance the systemic inflammatory response, often multiplied by inducing continuous generation of PGE_2_ as well as other prostaglandins. PGE_2_ can increase the transactivation of NF-*κ*B and further increase the generation of proinflammatory cytokines via a positive feedback mechanism [29, 30]. It is an effective therapeutic strategy for melioidosis to subsequently reduce PGE_2_ production via COX-2 inhibition [31].

Until now, there has been no direct experimental data to confirm that the expression of* Tnfaip3*,* Zbp1*,* Ccl9*,* Ifnb1*,* Ccl5*, and* Ifi202b* is upregulated in macrophages infected with* B. pseudomallei*. However, studies using similar stimulation but in different cell lines demonstrated the upregulation of 6 of the 10 DEGs identified in the current study.* Tnfaip3* (also called* A20*) was first defined as a* Tnf*-inducible gene in human umbilical vein endothelial cells, involved in inhibiting NF-*κ*B signaling and inflammation, and it can protect cells from* Tnfα*-induced cytotoxicity [32, 33]. Overexpression of* A20* can inhibit* Tnfα*-induced apoptosis and NF-*κ*B activation [34]. ZBP1, the product of DEG* Zbp1*, was originally described as a protein that was highly upregulated in response to lipopolysaccharide and IFN-*γ* stimulation in macrophages [35].

During osteoclast differentiation of rat bone marrow-derived mononuclear cells,* Ccl9* mRNA was highly upregulated. And under TNF superfamily member stimulation, the expression of* Ccl9* mRNA was increased by over 100-fold [36–38]. Downregulation of* Ccl9* chemokines by fusion protein BCR-ABL could help leukemic cells evade the immune system [39]. In primary microglia stimulated with lipopolysaccharide (10 ng/mL), the expression of inflammatory genes, including* Tnfα* and* Ccl5*, was significantly upregulated [37]. In addition, the expression of* Ifnb1* in the lymphocytes of melioidosis patients was upregulated compared with that in other sepsis cases [40].* Ifi202b*, a member of the interferon- (*Ifn*-) inducible* Ifi200* gene family, encodes p202b, which is* Ifn*-inducible, and differs from p202a with only 7 of 445 amino acids. Expression of* Ifi202* mRNA can be detected in many kinds of adult mouse tissues [41]. Expression of* Ifi202* is upregulated by both type I and II interferons via* Ifn*-responsive cis-elements, termed* Ifn*-stimulated response elements, in the promoter region [42].There are few reports regarding the expression of* Nfkbia* and* Nfkbie* during bacterial infection; however, genetic variations within* Nfkbia* and* Nfkbie* have been shown to influence susceptibility to invasive pneumococcal disease [4]. In the study, 10 DEGs were validated by qRT-PCR, and these DEGs were all immune associated. However, further studies are necessary to propose the hypothesis that tries to include some/all of DEGs into the known molecular and transcriptomic network of* B. pseudomallei*-infected macrophages.

In the study, we found that the number of DEGs from R8 versus R0 comparison was much less than that of R4 versus R0 and R11 versus R0 comparison. Only one biological replicate at each time point may be responsible for this result because only one biological replicate will lead to poor sensitivity or specificity in differential expression testing. Multiple biological replicates not only can decrease the background differences among samples, but also can measure the degree of variation within the same group and eliminate intragroup errors. By calculating the correlation among the samples in the same group, abnormal samples can be found and excluded. To sum up, multiple biological replicates potentially improve the reliability of results and should be incorporated for future RNA-Seq studies. Despite the limitation, our RNA-Seq findings have largely been validated by qPCR, which can deepen the understanding of the interactive mechanism between* B. pseudomallei* and host cells.

## 5. Conclusions

In this study, we identified 4668 DEGs in RAW264.7 cells during the early course of* B. pseudomallei* infection. After STEM clustering and KEGG enrichment analysis, 10 immune process-associated genes in RAW264.7 macrophage cells were shown to be upregulated in response to* B. pseudomallei* infection. Our findings can improve the understanding of the biological function of the ten immune process-associated genes during* B. pseudomallei* infection and are valuable for the prophylaxis and treatment of melioidosis. However, further functional analysis of the ten immune process-associated genes is still required and warranted.

## Figures and Tables

**Table 1:** Data summary of RNA-Seq experiments.

Sample	Raw reads	Clean reads	Q20	Mapping rate to reference genome
R0	33,995,054	33,755,316	97.15%	88.16%
R4	32,631,376	32,415,278	97.23%	88.98%
R8	31,079,714	30,803,170	96.85%	87.71%
R11	31,307,946	31,058,640	97.03%	87.12%

**Table 2:** DEGs from eight selected immune process-associated pathways in profile 25.

Pathway (Pathway ID)	Symbol
Cytokine-cytokine receptor interaction (ko04060)	*Ccl5, Ccl9, Ccr1, Ccr2, Clcf1, Ifnb1, Il11, Osm, Tnfα, Vegfa*
Cytosolic DNA-sensing pathway (ko04623)	*Ccl5, Ifnb1, Ifi202b, Nfkbia, Zbp1*
NOD-like receptor signaling pathway (ko04621)	*Ccl5, Nfkbia, Tnfα, Tnfaip3*
TNF signaling pathway (ko04668)	*Ccl5, Nfkbia, Tnfα, Tnfaip3, Ptgs2*
Chemokine signaling pathway (ko04062)	*Bcar1, Ccr1, Ccr2, Ccl5, Ccl9, Nfkbia*
NF-kappa B signaling pathway (ko04064)	*Nfkbia, Ptgs2, Tnfα, Tnfaip3*
Toll-like receptor signaling pathway (ko04620)	*Ccl5, Ifnb1, Nfkbia, Tnfα*
T cell receptor signaling pathway (ko04660)	*Nfkbia, Nfkbie, Tnfα*

## Data Availability

The raw RNA-Seq data of four samples was deposited into Sequence Read Archive (SRA) of National Center for Biotechnology Information (NCBI) with the accession number SRP115993.

## Supplementary Materials

Supplementary 1 KEGG pathway enrichment analysis of DEGs in profiles 7, 6, 4, 3, 18, and 16.

Supplementary 2 List of primers used in qRT-PCR validation.

Supplementary 3 DEGs in R4 versus R0 comparison.

Supplementary 4 DEGs in R8 versus R0 comparison.

Supplementary 5 DEGs in R11 versus R0 comparison.
