# Supplementary material for: Upregulation of Immune Process-Associated Genes in RAW264.7 Macrophage Cells in Response to Burkholderia pseudomallei Infection
Source: Biomed Res Int. 2018 Jun 4;2018:1235097. doi: 10.1155/2018/1235097 (PMC6008862; doi:10.1155/2018/1235097)
Supplement: Supplementary 1 — KEGG pathway enrichment analysis of DEGs in profiles 7, 6, 4, 3, 18, and 16. [file 1235097.f1.doc]

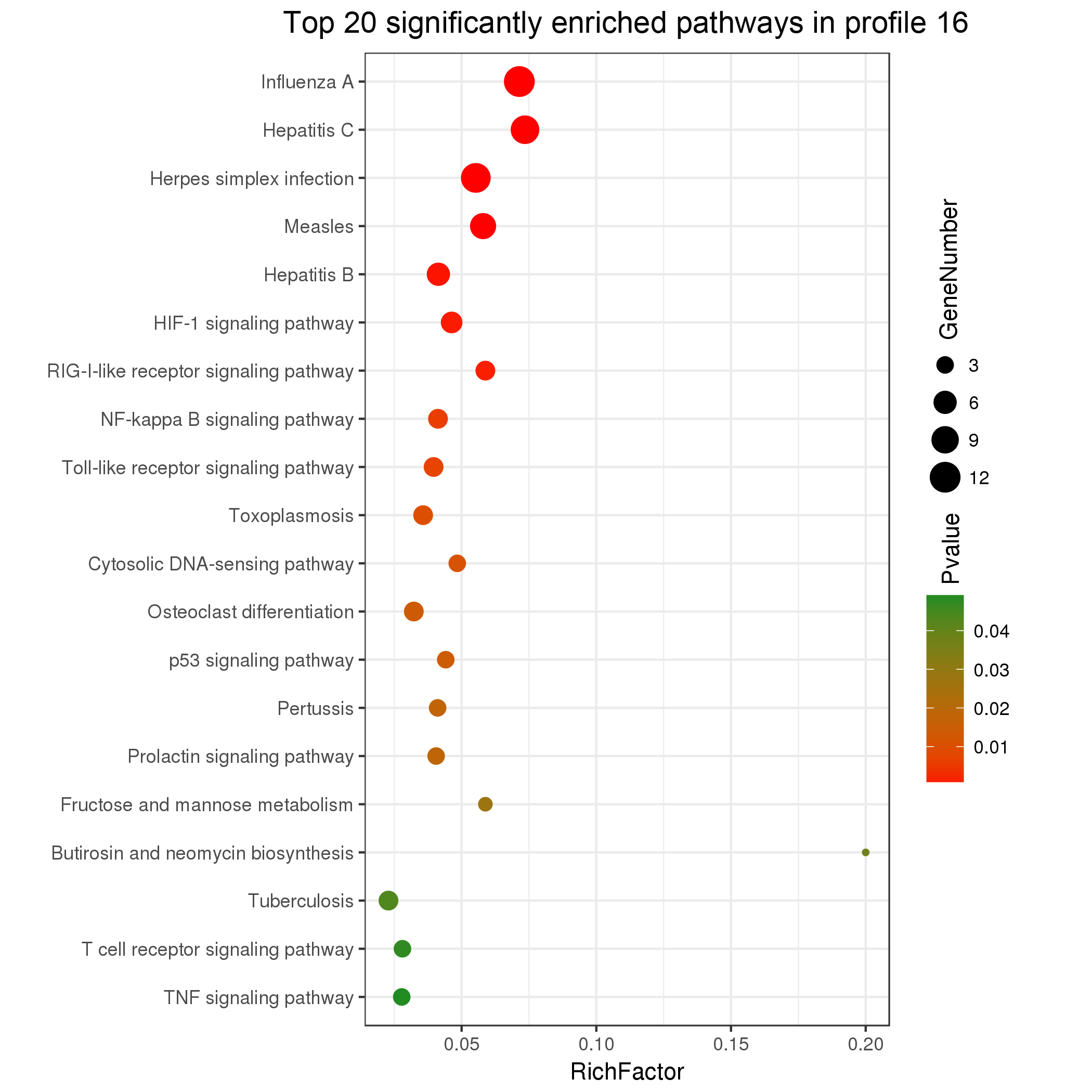

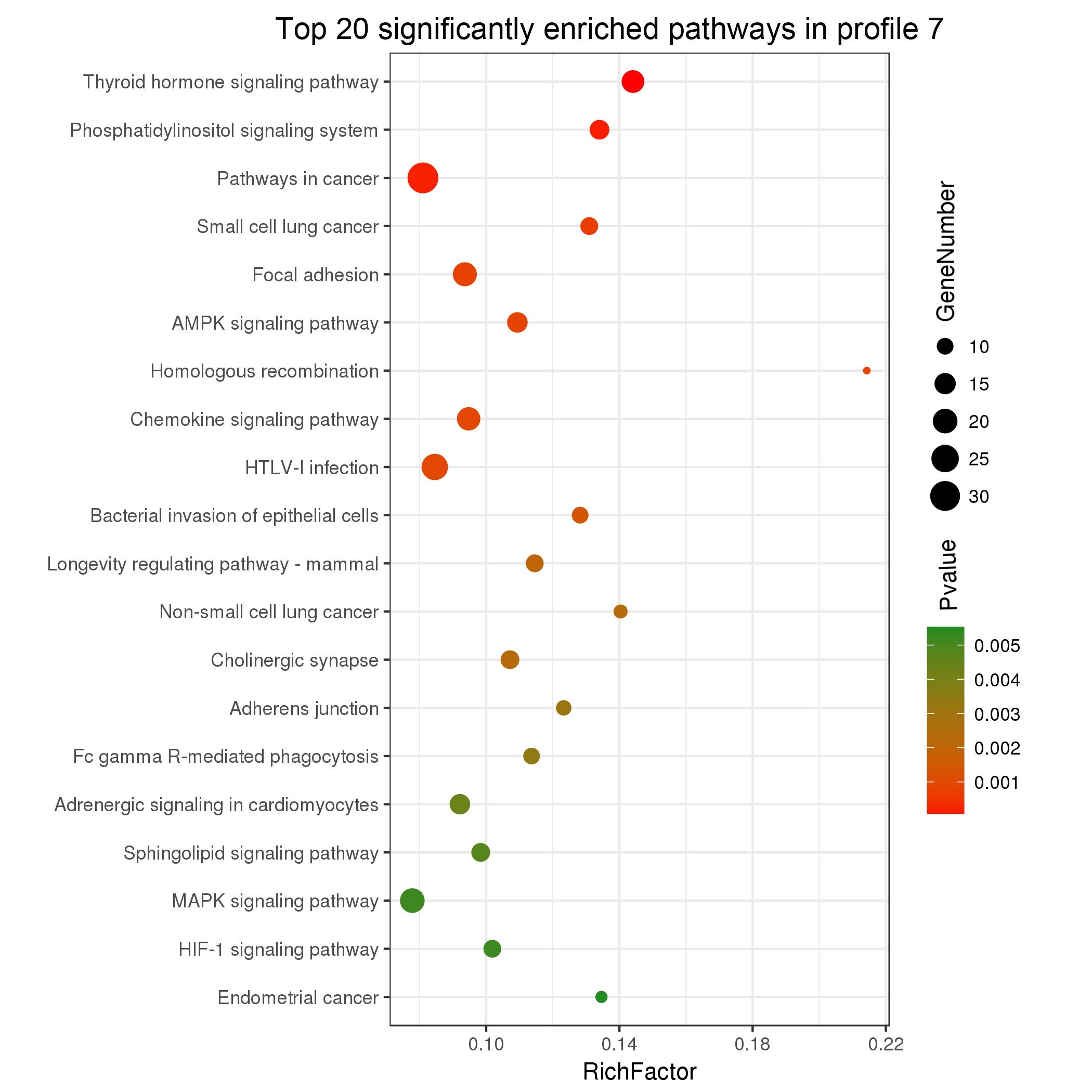

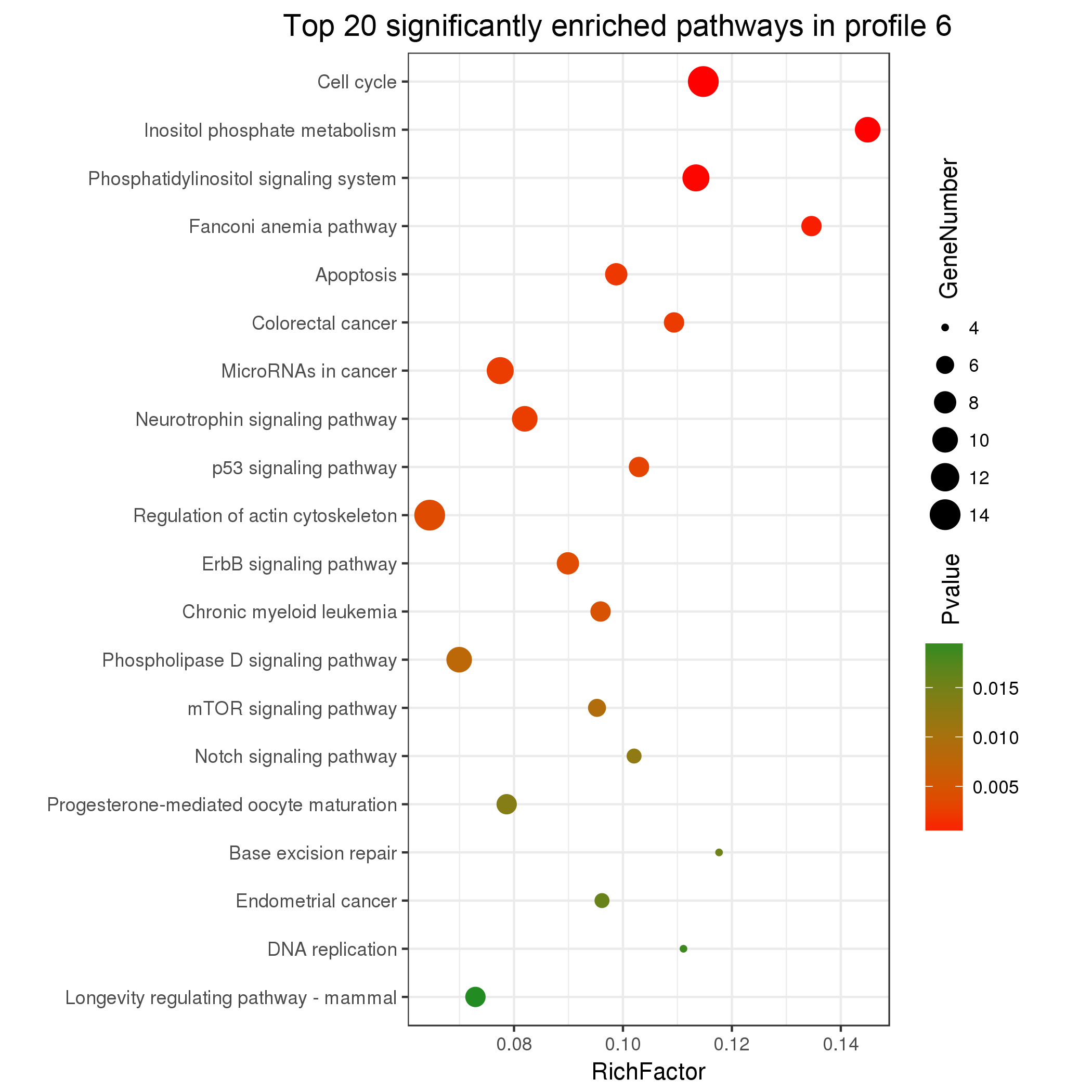

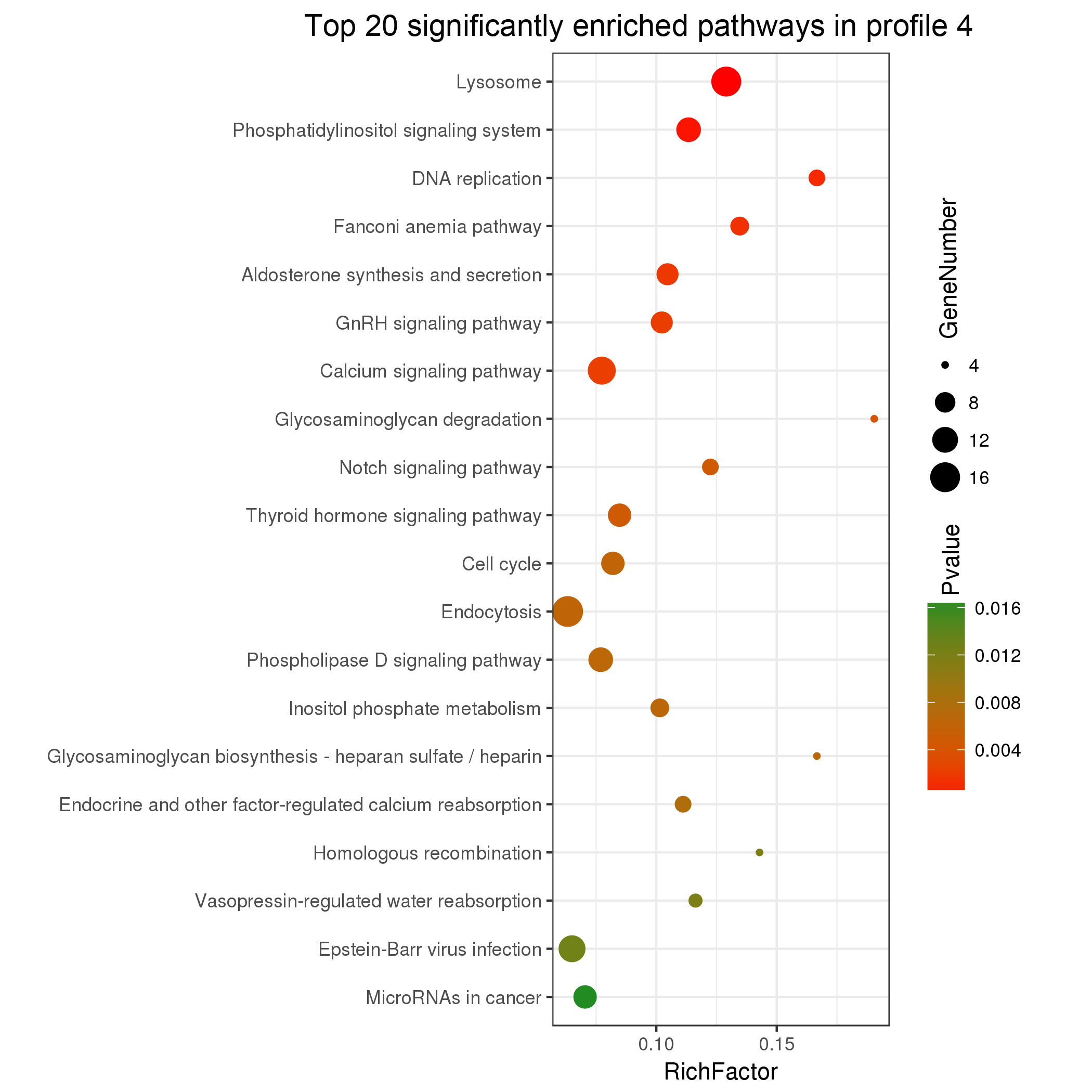

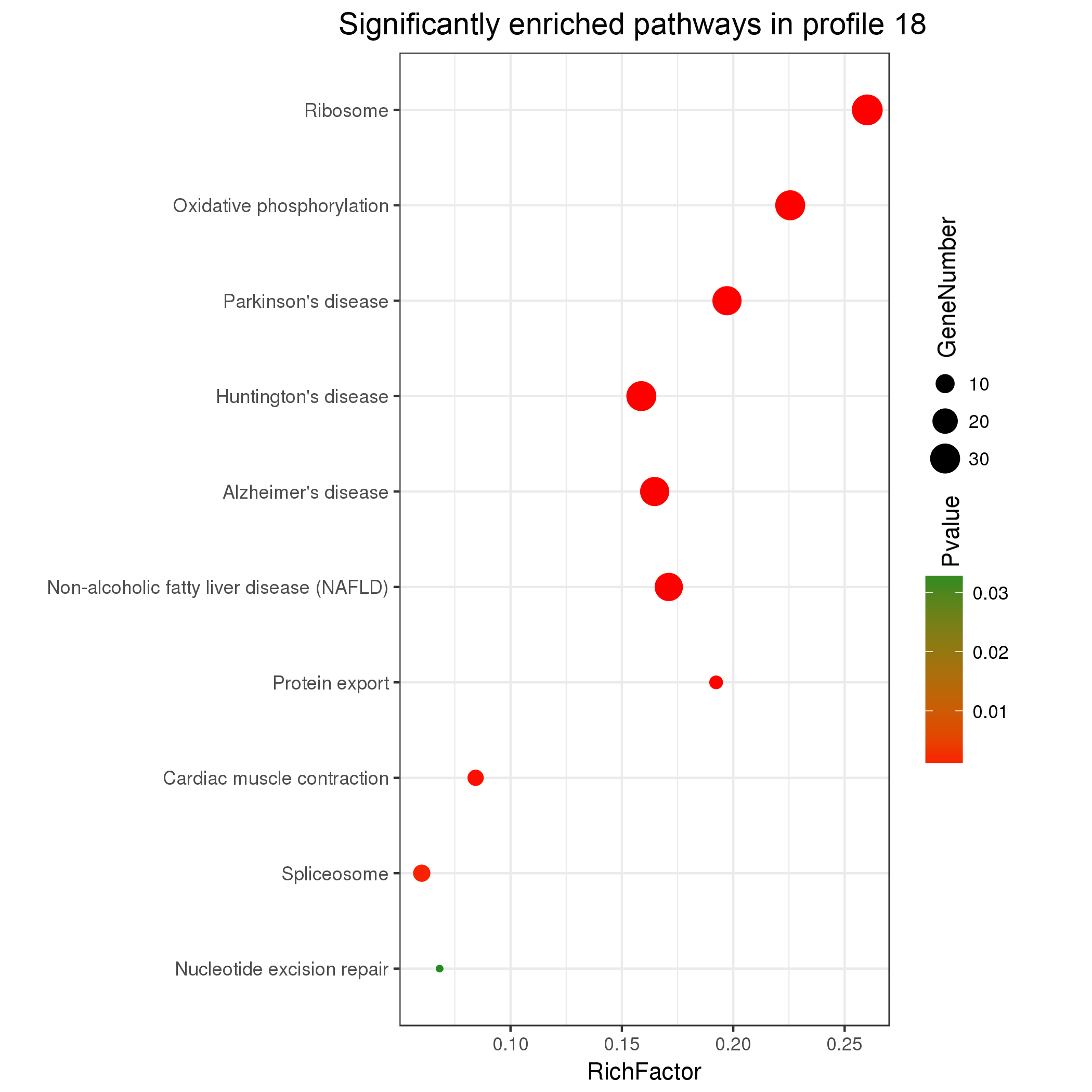

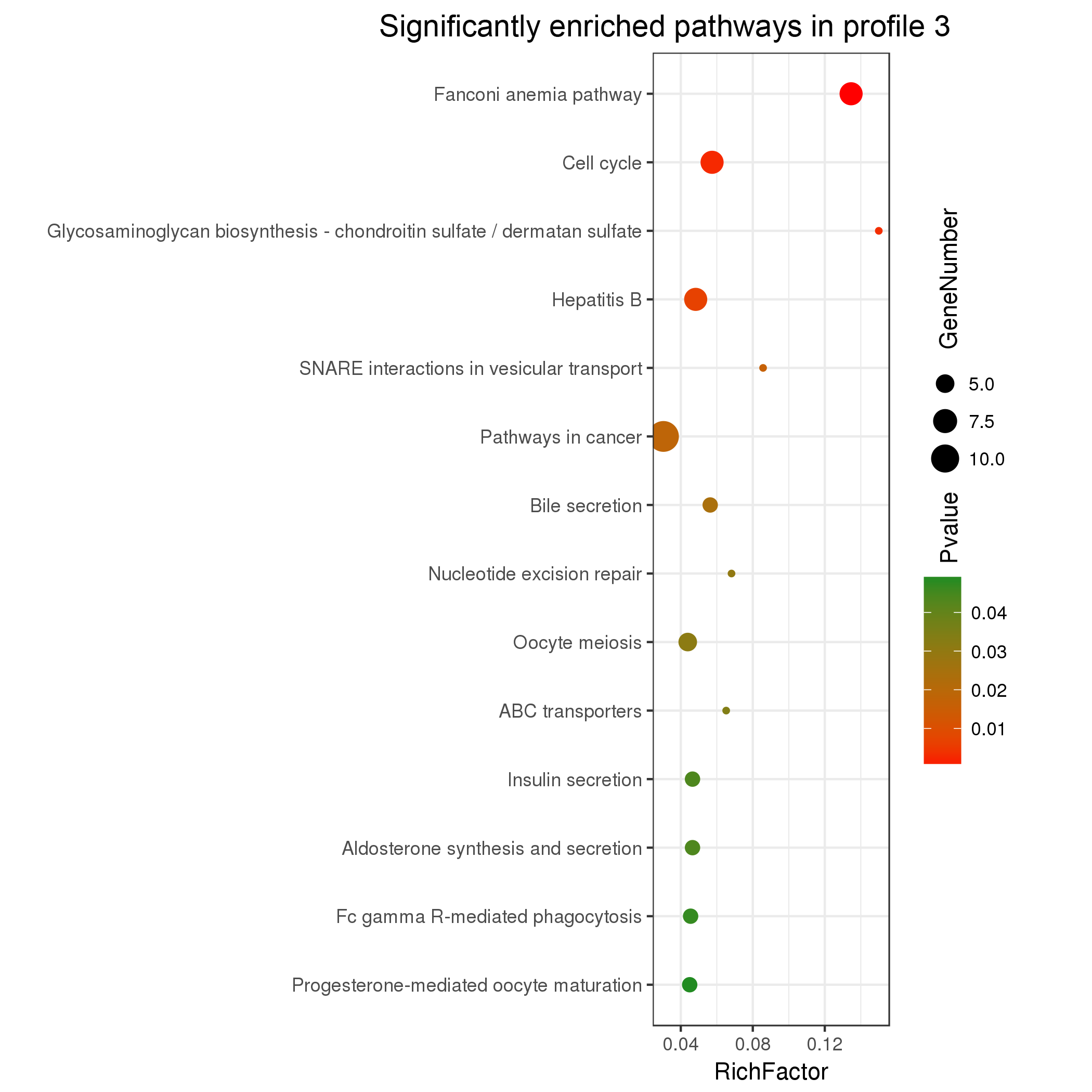


Supplementary Figure 1：KEGG pathway enrichment analysis of DEGs in profile7, 6, 4, 3, 18 and 16. Y-axis represented pathways, and X-axis represented rich factor (rich factor =the number of DEGs enriched in a pathway/the number of all genes annotated to the pathway).Color and size of each bubble represented enrichment significance and amount of DEGs enriched in a pathway, respectively.
