# Supplementary material for: Upregulation of Immune Process-Associated Genes in RAW264.7 Macrophage Cells in Response to Burkholderia pseudomallei Infection
Source: Biomed Res Int. 2018 Jun 4;2018:1235097. doi: 10.1155/2018/1235097 (PMC6008862; doi:10.1155/2018/1235097)
Supplement: Supplementary 2 — List of primers used in qRT-PCR validation. [file 1235097.f2.docx]

Supplementary Table 1: List of primers used in qRT-PCR validation

| Gene Symbol | Primer Sequence（5’-3’） | NCBI Reference Sequence |
| --- | --- | --- |
| *Ifnb1* | F:CAGCTCCAAGAAAGGACGAAC  R:GGCAGTGTAACTCTTCTGCAT | NM_010510.1 |
| *Ccl5* | F:TTGTCACTCGAAGGAACCGC  R:TACTGAGTGGCATCCCCAAG | NM_013653.3 |
| *Ccl9* | F:GCCCAGATCACACATGCAAC  R:GAACTCTCCGATCACTGGGG | NM_011338.2 |
| *Tnfα* | F:ATGGCCTCCCTCTCATCAGT  R:TTTGCTACGACGTGGGCTAC | NM_013693.3 |
| *Nfkbia* | F:GGGTGATTCGGCTGTTGTCT R:CCACTGAACACCTGGCTCTT | NM_010907.2 |
| *Ptgs2* | F:TGCTGGTGGAAAAACCTCGT R:AAAACCCACTTCGCCTCCAA | NM_011198.4 |
| *Tnfaip3* | F:GCCCAGTCTGTAGTCTTCGG R:TTGTTCAGCCATGGTCCTCG | NC_000006.12 |
| *Zbp1* | F:GCTATGACGGACAGACGTGG R:GTTGACCGGATTGTGCTGAC | NM_001139519.1 |
| *Ifi202b* | F:GCTGTACATCTGATAACACCTTCA R:TCCAGGAGAGGCTTGAGGTT | NM_008327.2 |
| *Nfkbie* | F:CTCCGGCAAACCACTGCTAT  R:GATCGGCTCTTCCTCGTCTG | NM_008690.4 |
| *Gapdh* | F:TGTGTCCGTCGTGGATCTGA  R:CCTGCTTCACCACCCTTCTTGA | NM_008084.3 |
